# Supplementary material for: Leveraging Podcasts to Introduce Medical Students to the Broader Community of Health Care Professionals
Source: MedEdPORTAL. 2021 Oct 25;17:11191. doi: 10.15766/mep_2374-8265.11191 (PMC8542682; doi:10.15766/mep_2374-8265.11191)
Supplement: Supplementary file 1 — Podcast Interview Guide.docxPodcast - Nurse Practitioner.mp3Podcast - Occupational Therapist.mp3Podcast - Social Worker.mp3Podcast - Speech-Language Pathologist.mp3Facilitator Guide.docxIPEC Competency Self-Assessment.docxInterprofessional Clinical Conversations Framework.pptx [file mep_2374-8265.11191-s001.zip › F. Facilitator Guide.docx]

**Title:** Leveraging Podcasts to Introduce Medical Students to the Broader Community of Healthcare Professionals

**Session:**

Asynchronous Portion: 1-2 hours

Live Portion: 1 hour

**Learning Objectives**:

By the end of this session, learners will be able to:

1. Compare the distinct roles and responsibilities of the health professionals highlighted in the podcasts (Roles and Responsibilities)
2. Identify the value of learning from diverse health professionals in medical education (Educational Value)
3. Employ respectful language modeled in the podcasts and live follow-up conversation when communicating with or about interprofessional colleagues (Respectful Language)
4. Use the podcasts and live follow-up conversation to develop strategies to engage in conversations with interprofessional colleagues throughout clinical rotations (Interprofessional Communication Strategies)

**Participants:** Medical students (2^nd^ year students, transitioning to clinical rotations)

**Prework**

1. Complete IPEC Competency Self-Assessment Survey
2. Review at least 2 out of 4 podcasts with interprofessional interviews with colleagues from social work, occupational therapy, nursing, and speech-language pathology.
   1. Podcast with Nurse Practitioner (23:43)
   2. Podcast with Occupational Therapist (25:40)
   3. Podcast with Social Worker (38:06)
   4. Podcast with Speech-Language Pathologist (27:56)
3. Contribute at least 1 follow up question for interviewers for live session using editable Google Doc Form.
   1. Facilitators review questions prior to session with assistance from student volunteers who organized questions into themes and prioritized questions for session.

**Session Outline: Live Panel**

Introduction (10 minutes)

- Welcome students to the interprofessional healthcare community
  - Facilitators reference the prework from the asynchronous portion of the session and provide an overview of the three components of the live session
    - Introduction of the IPEC competencies and importance of IPE to physician training
    - Highlighting the Interprofessional Clinical Conversations framework to facilitate conversations with colleagues from other health professions
    - Panel discussion with interprofessional colleagues from Podcast
  - Facilitators briefly share something they learned by listening to the podcasts
    - Goal is to model that learning about and from interprofessional colleagues occurs not only in medical training, but throughout one’s career
  - Series of three zoom polls to start session
    - *Zoom® Poll 1:* Which podcasts did you listen to?
      - *Response Options (check all that apply):* Speech and Language Pathology, Social Work, Nursing, Occupational Therapy
    - *Zoom® Poll 2:* How comfortable do you feel engaging in clinical conversations with other health professionals?
      - *Response Options (select 1):* Not comfortable, Somewhat comfortable, Very comfortable
    - *Zoom® Poll 3:* How comfortable do you feel asking other health professionals for feedback?
      - *Response Options (select 1):* Not comfortable, Somewhat comfortable, Very comfortable

IPEC Competencies and Interprofessional Clinical Conversations Framework (10 minutes**)**

- Review the IPEC Competencies (5 minutes)
  - The Interprofessional Education Collaborative (IPEC) proposed 4 major competencies as a framework for interprofessional collaboration that are instrumental to patient and family-centered care, as well as contributions to and advocacy for community and population-oriented care.
  - The competencies focus on shared values and ethics across health professions, understanding roles and responsibilities of different health professionals, and learning practices for effective communication and team-based practice.
  - Today’s session will focus on the fundamental need to understand the unique roles and responsibilities of healthcare professionals and how these overlap with and differ from our roles and responsibilities as physicians. Understanding where expertise lies is critical to leveraging it for patients.
- Introduce “*Student Framework: Continued Conversations around Interprofessional Collaboration”* (5 minutes)
  - To realize the IPEC competencies, it is helpful to engage in frequent interprofessional conversations. These may be specific to patient care but may also be more focused on expanding your knowledge of other health professions or soliciting their feedback.
    - We have created a brief framework to support you in facilitating your own interprofessional conversations while on upcoming clinical clerkships.
    - The goal is that this activity will help you feel comfortable engaging interprofessional colleagues in your medical education and development as a physician.
  - Brief review of “*Student Framework: Continued Conversations around Interprofessional Collaboration”*
    - The framework supplies points to reflect on prior to the conversation:
      - From your perspective, was the collaboration successful? What went well? What could have been improved?
      - Are their specific aspects around which you want specific feedback from your interprofessional colleague?
      - Did you learn anything new about the health care profession through the collaboration? Do you have outstanding questions?
    - In the conversation itself, you will have the opportunity to jointly reflect on the interaction from the perspective of the IPEC competencies. Questions you might explore include:
      - How are your values similar or different from interprofessional colleagues? How might these similarities (differences) enhance patient care?
      - How did these interactions impact your understanding of the roles and responsibilities of interprofessional colleagues? How do other team members complement your own abilities?
      - Can you identify any moments of interprofessional conflict? How were these handled?
      - How does language used when speaking to or about interprofessional colleagues contribute to or detract from team functioning?
    - To initiate and ensure productive conversations with Interprofessional Colleagues, it helps to:
      - Approach colleagues with curiosity about their profession and perspective
      - Clearly express your desire to learn and improve
      - Identify a mutually agreeable time; find a place that will minimize interruptions

Interview with interprofessional colleagues from the podcast (30 minutes)

- Goal is to facilitate a conversation around how to best leverage expertise of other professionals, building from the podcast interviews. Also serves as a venue to answer follow up questions generated by medical students from the asynchronous material.
- Introduction of interprofessional colleagues participating in live session
  - Each colleague introduces self and opens with a story about a time when they collaborated with a medical student or other trainee with a focus on identifying what went well and what could have gone better.
- Facilitator Questions
  - How would you respond to a student approaching you for feedback?
  - What could the student do that might make the conversation more or less successful?
  - Identify strategies that they have employed to facilitate improved communication with students and other trainees (residents, fellows, etc.)
- Q&A with facilitators posing follow up questions from students using those submitted on Google form and curated by medical student leaders; also allow option for live submission of questions via the Zoom® Chat box.

Wrap Up (5 minutes)

- Thank interprofessional colleagues for joining us live and on the podcast to offer their unique experience and expertise
- Reiterate the 4 main IPEC competencies and orient the students to the fact that they will be returning to these throughout their pre-clinical and clinical coursework
- Emphasize that the IPEC competencies can serve as a framework to guide their own learning outside of these intentional moments, as interprofessional collaboration is a thread that will run continuously throughout their medical education and professional career
  - Reinforce the Interprofessional Clinical Conversations framework as a structure that will help them have these conversations throughout their clinical experience

Post-Session Assessment Survey – Qualtrics link shared via QR code with link to IPEC self-assessment in final slide shared in Zoom® shared window.
