# Supplementary material for: Leveraging Podcasts to Introduce Medical Students to the Broader Community of Health Care Professionals
Source: MedEdPORTAL. 2021 Oct 25;17:11191. doi: 10.15766/mep_2374-8265.11191 (PMC8542682; doi:10.15766/mep_2374-8265.11191)
Supplement: Supplementary file 1 — Podcast Interview Guide.docxPodcast - Nurse Practitioner.mp3Podcast - Occupational Therapist.mp3Podcast - Social Worker.mp3Podcast - Speech-Language Pathologist.mp3Facilitator Guide.docxIPEC Competency Self-Assessment.docxInterprofessional Clinical Conversations Framework.pptx [file mep_2374-8265.11191-s001.zip › G. IPEC Competency Self-Assessment.docx]

**Appendix G – IPEC Competency Self-Assessment**

Adapted by the authors from the IPEC Competency Self-Assessment Tool (Version 3)^1,2^

**INSTRUCTIONS**: Based on your education or experience in the health care environment, select/circle the number that corresponds with your level of agreement or disagreement on each item.

|  | **Strongly Disagree** | **Disagree** | **Neither Agree nor Disagree** | **Agree** | **Strongly Agree** |
| --- | --- | --- | --- | --- | --- |
| 1. I am able to choose communication tools and techniques that facilitate effective team interactions. | 1 | 2 | 3 | 4 | 5 |
| 1. I am able to place the interests of patients at the center of interprofessional health care delivery. | 1 | 2 | 3 | 4 | 5 |
| 1. I am able to engage other health professionals in shared problem-solving appropriate to the specific care situation. | 1 | 2 | 3 | 4 | 5 |
| 1. I am able to respect the privacy of patients while maintaining confidentiality in the delivery of team-based care. | 1 | 2 | 3 | 4 | 5 |
| 1. I am able to inform care decisions by integrating the knowledge and experience of other professions appropriate to the clinical situation. | 1 | 2 | 3 | 4 | 5 |
| 1. I am able to embrace the diversity that characterizes the health care team. | 1 | 2 | 3 | 4 | 5 |
| 1. I am able to apply leadership practices that support effective collaborative practice. | 1 | 2 | 3 | 4 | 5 |
| 1. I am able to respect the cultures and values of other health professions. | 1 | 2 | 3 | 4 | 5 |
| 1. I am able to engage other health professionals to constructively manage disagreements about patient care. | 1 | 2 | 3 | 4 | 5 |
| 1. I am able to develop a trusting relationship with other team members. | 1 | 2 | 3 | 4 | 5 |
| 1. I am able to use strategies that improve the effectiveness of interprofessional teamwork and team-based care. | 1 | 2 | 3 | 4 | 5 |
| 1. I am able to demonstrate high standards of ethical conduct in my contributions to team-based care. | 1 | 2 | 3 | 4 | 5 |
| 1. I am able to use available evidence to inform effective teamwork and team-based practices. | 1 | 2 | 3 | 4 | 5 |
| 1. I am able to act with honesty and integrity in relationships with other team members. | 1 | 2 | 3 | 4 | 5 |
| 1. I am able to understand the responsibilities and expertise of other health professions. | 1 | 2 | 3 | 4 | 5 |
| 1. I am able to maintain competence in my own profession appropriate to my level of training. | 1 | 2 | 3 | 4 | 5 |
| 1. Listening to the podcast interviews with provider from other health care professions enhanced my learning in this session. | 1 | 2 | 3 | 4 | 5 |
| 1. The panel featuring providers from other health care professions enhanced my learning in this session | 1 | 2 | 3 | 4 | 5 |

*Question:* I was familiar with the Interprofessional Education Collaborative (IPEC) competencies prior to this session.

*Answer Options:* True False

*Question:* Please describe one thing you learned about interprofessional collaboration during this session.

Answer Options: Free text reply

REFERENCES

1. Lockeman KS, Dow AW, Randell AL. Validity evidence and use of the IPEC Competency Self-Assessment, Version 3. *J Interprof Care*. 2019. doi:10.1080/13561820.2019.1699037

2. Dow AW, Diazgranados D, Mazmanian PE, Retchin SM. An exploratory study of an assessment tool derived from the competencies of the interprofessional education collaborative. *J Interprof Care*. 2014;28(4):299-304. doi:10.3109/13561820.2014.891573
