# Supplementary material for: Leveraging Podcasts to Introduce Medical Students to the Broader Community of Health Care Professionals
Source: MedEdPORTAL. 2021 Oct 25;17:11191. doi: 10.15766/mep_2374-8265.11191 (PMC8542682; doi:10.15766/mep_2374-8265.11191)
Supplement: Supplementary file 1 — Podcast Interview Guide.docxPodcast - Nurse Practitioner.mp3Podcast - Occupational Therapist.mp3Podcast - Social Worker.mp3Podcast - Speech-Language Pathologist.mp3Facilitator Guide.docxIPEC Competency Self-Assessment.docxInterprofessional Clinical Conversations Framework.pptx [file mep_2374-8265.11191-s001.zip › H. Interprofessional Clinical Conversations Framework.pptx]

## Slide 1
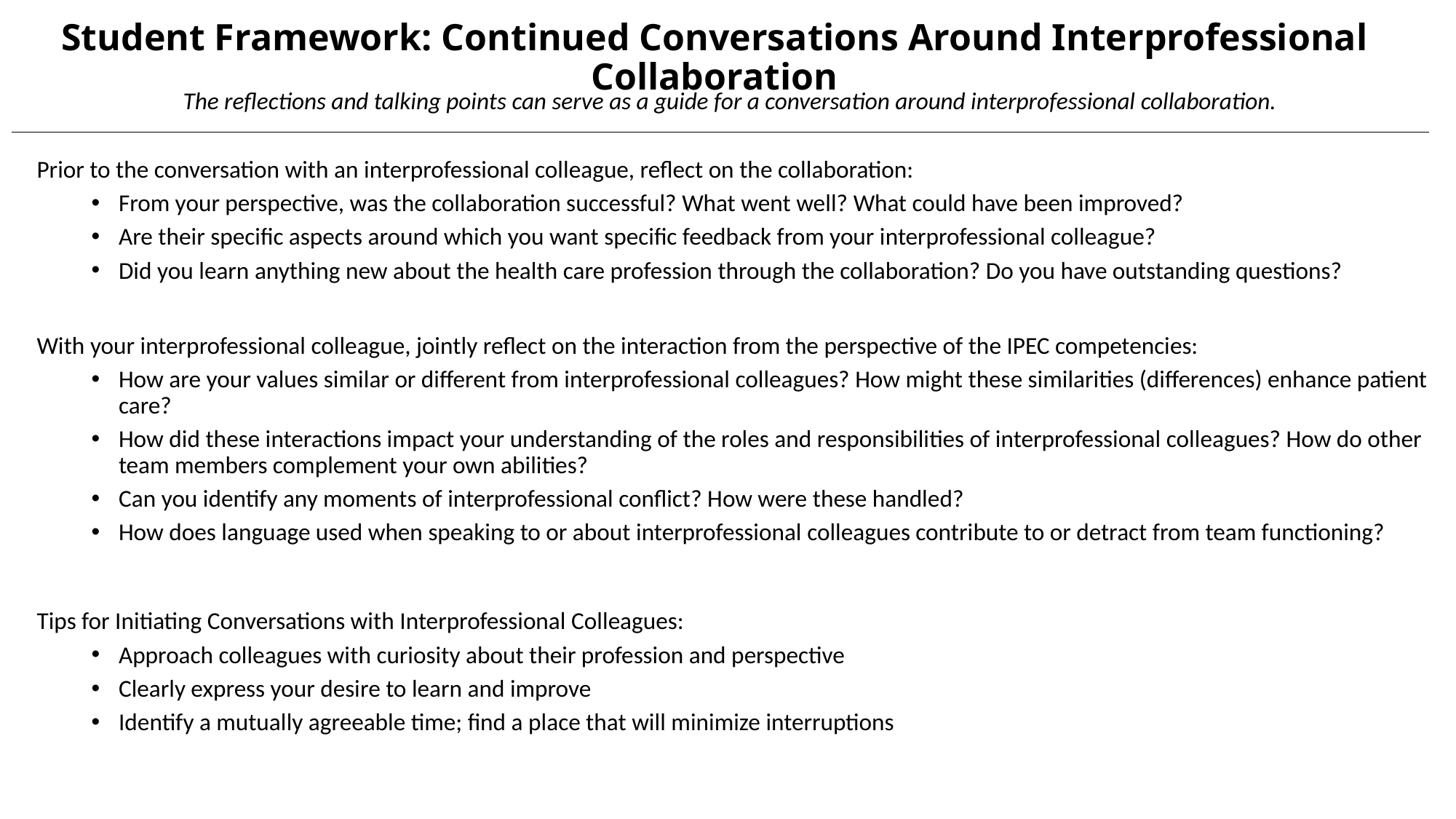

# Student Framework: Continued Conversations Around Interprofessional Collaboration
The reflections and talking points can serve as a guide for a conversation around interprofessional collaboration.
Prior to the conversation with an interprofessional colleague, reflect on the collaboration:
From your perspective, was the collaboration successful? What went well? What could have been improved?
Are their specific aspects around which you want specific feedback from your interprofessional colleague?
Did you learn anything new about the health care profession through the collaboration? Do you have outstanding questions?
With your interprofessional colleague, jointly reflect on the interaction from the perspective of the IPEC competencies:
How are your values similar or different from interprofessional colleagues? How might these similarities (differences) enhance patient care?
How did these interactions impact your understanding of the roles and responsibilities of interprofessional colleagues? How do other team members complement your own abilities?
Can you identify any moments of interprofessional conflict? How were these handled?
How does language used when speaking to or about interprofessional colleagues contribute to or detract from team functioning?
Tips for Initiating Conversations with Interprofessional Colleagues:
Approach colleagues with curiosity about their profession and perspective
Clearly express your desire to learn and improve
Identify a mutually agreeable time; find a place that will minimize interruptions
