# Supplementary material for: Leveraging Podcasts to Introduce Medical Students to the Broader Community of Health Care Professionals
Source: MedEdPORTAL. 2021 Oct 25;17:11191. doi: 10.15766/mep_2374-8265.11191 (PMC8542682; doi:10.15766/mep_2374-8265.11191)
Supplement: Supplementary file 1 — Podcast Interview Guide.docxPodcast - Nurse Practitioner.mp3Podcast - Occupational Therapist.mp3Podcast - Social Worker.mp3Podcast - Speech-Language Pathologist.mp3Facilitator Guide.docxIPEC Competency Self-Assessment.docxInterprofessional Clinical Conversations Framework.pptx [file mep_2374-8265.11191-s001.zip › A. Podcast Interview Guide.docx]

**Session Outline 1: Asynchronous Portion**

We recorded podcast interviews with interprofessional colleagues from social work, occupational therapy, nursing, and speech-language pathology

Interviews were pre-recorded using Zoom^®^ with interprofessional colleagues who volunteered to participate. The following questions were generated by faculty and medical students and provided an outline for each interview:

- Why/how did you decide on your profession?
  - What is your training like? Is there an internship or advanced training?
  - Are there roles to your profession/job that most people don’t know you do? What is the scope of practice available to your profession?
  - What is the most fulfilling part of your profession/job? Is there any part of your profession/job that you dislike?
  - What is your most favorite and least favorite part of your profession/job?
  - How to people in your profession interact with physician teams in the hospital setting?
  - How do people in your profession interact with physician teams in the ambulatory setting?
  - Can you give us an example of collaborative patient care that went well- and why?
  - Can you give us an example of collaborative patient care that did not go well- and why?
  - Can you tell us one aspect or fact about your profession you wish more people (or physicians) knew?
  - What advice would you give to students as to the most effective manner to initiate a conversation about patient care, or call for a consult?
  - If you were to create your ideal physician, what key characteristics would that person have?
  - Can you tell us about a memorable patient encounter?
