# Supplementary material for: Leveraging Podcasts to Introduce Medical Students to the Broader Community of Health Care Professionals
Source: MedEdPORTAL. 2021 Oct 25;17:11191. doi: 10.15766/mep_2374-8265.11191 (PMC8542682; doi:10.15766/mep_2374-8265.11191)
Supplement: Supplementary file 1 — Podcast Interview Guide.docxPodcast - Nurse Practitioner.mp3Podcast - Occupational Therapist.mp3Podcast - Social Worker.mp3Podcast - Speech-Language Pathologist.mp3Facilitator Guide.docxIPEC Competency Self-Assessment.docxInterprofessional Clinical Conversations Framework.pptx [file mep_2374-8265.11191-s001.zip › Educational_Summary_Report_11191.pdf]

MedEdPORTAL® The AAMC Journal of Teaching and Learning Resources

Original Publication

Open Access

Leveraging Podcasts to Introduce Medical Students to the Broader Community of Health Care Professionals

Kelsey A. Miller, MD, EdM*, Tamra Keeney, DPT, PhD, Allison Fialkowski, BSN, Sanjana Srinivasan, Tara A. Singh, MD, Jennifer Kesselheim, MD, MEd, Susan Farrell, MD, EdM, Cynthia Cooper, MD, Celeste S. Royce, MD

*Corresponding author: kelsey.miller@childrens.harvard.edu

Abstract

Introduction: Safe, patient-centered, and cost-effective care requires effective collaboration within interprofessional teams. Education programs for health care professionals are often siloed, providing students with limited interprofessional education (IPE) opportunities to learn from, with, and about other professions. Podcasts offer a novel approach to facilitate IPE, allowing for asynchronous conversations with interprofessional colleagues. **Methods:** We developed four podcasts with various health care professionals for 135 preclinical medical students preparing to transition into clinical rotations. The podcasts were coupled with an hour-long interactive session with the podcast interviewees conducted via videoconference. The curriculum explored the distinct education paths, roles, and responsibilities of various health care disciplines. Strategies for communicating effectively with and learning from interprofessional team members were emphasized. **Results:** There were 197 unique downloads of the podcasts, and 95 students attended the interactive session. Most students reported that the podcasts and follow-up live session enhanced their learning (100% and 98% of students who completed the postcurriculum survey, respectively). Responses to the postcurriculum survey revealed students learned strategies for engaging in productive interprofessional conversations, the importance of leveraging the distinct roles and responsibilities of diverse health professionals, the value of learning from other health professionals, and the use of respectful language. **Discussion:** This IPE curriculum built around podcasts enhances medical student learning and represents an innovative approach to improving access to IPE in a virtual learning environment. This modality can be adapted to meet the needs of a wide spectrum of learners and can be coupled with in-person learning.

Keywords
Podcast, Interprofessional Education, Preclinical Medical Curriculum, Nurse/Nurse Practitioner, Occupational Therapist, Physical Therapist, Multimedia, Online/Distance Learning, Virtual Learning

Educational Objectives

By the end of this session, learners will be able to:

1. Compare the distinct roles and responsibilities of the health professionals highlighted in the podcasts.
2. Identify the value of learning from diverse health professionals in medical education.
3. Employ respectful language modeled in the podcasts and live follow-up conversation when communicating with or about interprofessional colleagues.
4. Use the podcasts and live follow-up conversation to develop strategies to engage in conversations with interprofessional colleagues throughout clinical rotations.

Citation:
Miller KA, Keeney T, Fialkowski A, et al. Leveraging podcasts to introduce medical students to the broader community of health care professionals. *MedEdPORTAL*. 2021;17:11191.
https://doi.org/10.15766/mep_2374-8265.11191

Introduction

Patient-centered care is increasingly emphasized in the current health care climate, with team-based care as a cornerstone for ensuring safe care delivery.[1] Yet medical students may lack a clear understanding of the expertise and contributions of various health care professionals on interprofessional health care teams.[2]

Interprofessional education (IPE) has been identified as paramount to increasing collaboration in health care, reducing widespread diagnostic error, and lowering rates of preventable mortality and morbidity. The Institute of Medicine has tasked education institutions to prepare a workforce capable of

Copyright © 2021 Miller et al. This is an open-access publication distributed under the terms of the Creative Commons Attribution license.

practicing health care to the full scope of its expertise by using a cooperative effort between professions.[3-5] The Liaison Committee on Medical Education includes IPE as an element of curricular content required for accreditation for all medical schools.[6] Despite this, IPE programs remain largely elective and inconsistently implemented in medical education curricula.[7]

The Interprofessional Education Collaborative (IPEC) has identified core competencies to facilitate interprofessional collaborative practice, yet their application in education and clinical practice is a work in progress.[7] In recent years, the Association of American Medical Colleges has included the ability to "collaborate as a member of an interprofessional team" as one of its 13 Entrustable Professional Activities relevant to the training of medical students.[8] Despite the growing commitment to IPE, barriers to implementation persist, including limited financial resources and administrative support, rigid curricula, and high student workload.[9]

Within this background, podcasts are becoming a preferred teaching and learning modality at all training levels.[10,11] Ease of use and availability of asynchronous access to material contribute to the wide acceptance of this medium. Online delivery allows modules to be accessed by all students at any time, at any speed and frequency.[12] Notably, the IPEC report indicates that for prelicensure/precredentialing education, interprofessional competencies are ultimately best demonstrated through clinical learning situations.[7] Podcasts, as sources of storytelling, provide a novel tool for introducing these clinical vignettes, a tool that has become even more critical with the COVID-19 pandemic potentially limiting students' access to clinical exposure.

Considering the need for innovative educational IPE strategies, we leveraged podcasts to educate medical students about the roles and responsibilities of other health care professionals. The IPEC has cited *MedEdPORTAL* as a location for high-quality IPE teaching materials, including simulations, problem-based learning, and video-based education.[7,13-16] An IPE curriculum using podcasts as the primary educational modality has yet to be published in *MedEdPORTAL,* and examples of podcasts to deliver IPE are also limited in the broader literature.[17,18] We hypothesized that using podcasts to interview nonphysician health professionals, coupled with a live follow-up session with the interviewees, would provide an effective learning tool for students to develop an understanding of the roles of other health professions in team-based patient care.

Methods

Curricular Context for Implementation

We developed a two-part workshop for preclerkship medical students as part of the Transitions course intended to prepare students to move from the preclerkship learning environment into core clinical rotations. We did not require or assume that learners had prior experience in IPE. We developed this workshop in response to the limitations on clinical learning imposed due to the COVID-19 pandemic. This session's educational objectives sought to foster three of the four IPEC competencies: (1) understanding roles and responsibilities of different health professionals that students will encounter (Educational Objective 1), (2) valuing and respecting the expertise of a diverse health care team (Educational Objective 2) and (3) strategies for communicating with and learning from interprofessional team members (Educational Objectives 3 and 4).

Our curriculum consisted of asynchronous prework delivered through podcasts accessible to students at any time, as well as a synchronous interactive session held remotely through the Zoom videoconferencing platform.

Two fundamental principles guided the creation of this curriculum. First, we aimed to couple the benefits of asynchronous learning, namely, its flexibility and ability to support a flipped classroom, with the opportunity to engage in live curated follow-up discussion. Second, we strove to incorporate student voices to guide the content and structure of the learning experience.[19]

Steps to Implementation

Our curriculum development team consisted of members of the school's IPE faculty team (three physicians and one physical therapist), course directors for the Transitions course (two physicians), and a team of medical students recruited from the entire student body. We included both preclerkship students enrolled in the Transitions course and students currently participating in clerkships.

Podcast creation: Course directors identified colleagues to be interviewed for the podcasts. In selecting colleagues, we sought representation from various professional backgrounds in both inpatient and outpatient care contexts. We recruited professionals from speech-language pathology, nursing, social work, and occupational therapy. The Harvard Medical School Office of Educational Quality Improvement approved this project as an educational quality improvement project. Interviewees gave informed consent to participate and share their podcasts.

Copyright © 2021 Miller et al. This is an open-access publication distributed under the terms of the Creative Commons Attribution license.

Course faculty and students jointly generated questions to guide podcast interviews. Questions focused on roles, responsibilities, and collaboration within health care teams (Appendix A). We conducted four podcast interviews (24-38 minutes) via Zoom calls in September 2020. The audio recordings of the full interviews were transformed and edited into four resulting podcasts (Appendices B-E) using a free web-based audio-editing program, Audacity. Based on the interviews and input from podcast interviewees, the authors developed the Student Framework: Continued Conversations Around Interprofessional Collaboration card (Appendix H) to help students engage in future interprofessional conversations during upcoming clerkships.

Students gained access to the course website 1 day prior to the live session. We instructed students to listen to at least two of the four podcast interviews to allow for student self-directed learning based on interests. Students submitted follow-up questions for the live session with the podcast interviewees.

One physical therapist (Tamra Keeney) and one physician (Kelsey A. Miller) served as facilitators for the 1-hour online session. We developed a facilitator guide (Appendix F) that outlined the structure and provided key teaching points. The course administrator created and hosted the Zoom session, scheduled the meeting, generated audience polls for in-session use, and monitored participants' interactions. We provided the interprofessional interviewees with the facilitator guide and asked them to prepare answers to standardized questions for the discussion portion of the online session. The interviewees did not receive the students' submitted questions prior to the live session.

The primary focus for the live session was to encourage further conversation between students and the podcast interviewees about the roles of and communication strategies employed by interprofessional colleagues. The session mapped these themes to the IPEC core competencies and introduced the Student Framework card to encourage future learning from health professionals during clinical rotations.

Live Session Time Line

Introduction (10 minutes): Facilitators began the session by welcoming students to the interprofessional health care community and introducing the facilitators and interviewees. Facilitators referenced the prework completed using the podcasts and provided an overview of the three components of the live session: (1) introduction of three of the IPEC competencies and importance of IPE to physician training and clinical practice, (2) opportunity for continued discussion with the interprofessional colleagues featured in the podcasts, and (3) introduction of a framework for future conversations to empower continued exploration of interprofessional collaboration during clinical rotations.

To foster engagement in the session, we included three audience Zoom polls during the live session (Appendix F). The first poll identified which podcasts students had reviewed prior to the session. The second and third Zoom polls asked students to rate their comfort engaging in conversations and seeking feedback from interprofessional colleagues, respectively.

IPEC competencies and Student Framework card (10 minutes): The facilitators introduced the core IPEC competencies as a framework for interprofessional collaboration and as necessary for providing patient- and family-centered care. The facilitators underscored the importance of learning about the unique roles and responsibilities of other health care professionals to enable truly collaborative care. We introduced the Student Framework card (Appendix H) to structure future interprofessional conversations during students' upcoming clinical rotations. We concluded by providing strategies for dialogue, conversations, and feedback in busy clinical environments.

Interview with interprofessional colleagues featured in podcasts (30 minutes): The main portion of the session included a live discussion with the interprofessional colleagues featured in the podcasts. Interviewees began by telling a story about a prior collaboration with a medical student or other trainee. Interviewees then identified effective communication strategies for medical students to employ when working on interprofessional teams. The last half of the discussion focused on addressing questions submitted by students in response to the podcasts. Our curriculum development team student members (Sanjana Srinivasan and Allison Fialkowski) reviewed and selected the questions for the live panel. Students were also encouraged to submit additional questions in real time through the Zoom Chat function.

If the prerecorded podcasts were used, this discussion could be conducted with other health professionals. Interprofessional guests could begin with storytelling, move on to sharing strategies for effective communication, and end with answering students' questions related to the podcasts. To equip the interprofessional guests to answer these questions, access to the prerecorded podcasts and the students' questions would need to be provided before the live discussion.

Copyright © 2021 Miller et al. This is an open-access publication distributed under the terms of the Creative Commons Attribution license.

Wrap-up (5 minutes): We concluded by summarizing the IPEC competencies, reinforcing the role of IPE throughout preclinical and clinical coursework, and reiterating the role of the Student Framework card to facilitate continued interprofessional communication. We asked learners to complete a final postassessment survey including the IPEC Competency Self-Assessment[20,21] and additional evaluative questions (Appendix G) accessed via a QR code link on the final slide of the Zoom presentation.

Session Evaluation

Immediately prior to viewing the podcasts, students completed the IPEC Competency Self-Assessment tool (Appendix G).[20,21] This instrument asked students to self-assess on 16 statements derived from the IPEC competencies and grouped into two domains: interprofessional interaction and interprofessional values. Each statement received a possible score from 1 (lowest) to 5 (highest). We asked but did not require students to supply an anonymous identifier to link their results on the prepodcast self-assessment (pretest) to the survey planned for the completion of the live session (posttest). Immediately after the live session, we surveyed students using the same IPEC Competency Self-Assessment tool with one additional qualitative and three additional quantitative questions to evaluate the value and learning of the two-part workshop (Appendix G). We sent a reminder email to all participants 2 days after the session to increase the response rate.

Statistical Analysis

We used descriptive statistics to characterize the proportion of students accessing each podcast and the responses to the Zoom polls. The IPEC Competency Self-Assessment generated two scores: one for the interprofessional values domain and one for the interprofessional interaction domain. We compared changes in both scores from pretest to posttest using paired *t* tests for respondents who completed the survey at both time points and unmatched *t* tests to compare the full population of students who responded to either survey.

We then identified qualitative themes that emerged from student reflections on what they had learned during the workshop. Two authors (Kelsey A. Miller and Tamra Keeney) independently mapped student responses to the four Educational Objectives. The authors then compared the maps, and any discrepancies were discussed until agreement was reached on which Educational Objective(s) to assign that response.

While all components (listening to two podcasts, attending the live session, and completing the pre- and posttests) were technically required, there were no penalties for students who did not complete any or all components.

Results

A total of 135 students were enrolled in the Transitions course. The pretest was completed by 77 of the 135 students (57%). Prior to participating in the two-part workshop, students reported lower comfort with statements related to interprofessional interactions compared to statements related to interprofessional values (63% agreement for interprofessional interaction domain vs. 89% agreement for interprofessional values domain; Table 1).

There were 197 unique downloads of podcast episodes by 80 students. Average minutes delivered per podcast download ranged from 11.4 to 14.6 (Table 2). Ninety-five students signed into the required live session. Polling of these 95 students at the beginning of the session revealed that after listening to the podcasts, almost all felt somewhat (76 out of 95, 80%) or very comfortable (16 out of 95, 17%) engaging in clinical interactions with other health professionals. Similarly, the majority felt somewhat (50 out of 95, 53%) or very comfortable (20 out of 80, 25%) asking other health professionals for feedback.

The posttest was completed by 39 of the 95 students who attended the live session. The posttest revealed that 68% of respondents had not heard of the IPEC competencies prior to the workshop. Scores on the IPEC Competency Self-Assessment improved for both domains from pretest to posttest (Table 3), a finding consistent among all respondents and for the subset of 31 students who supplied an anonymous identifier to match pretest and posttest data. All respondents (100%) agreed or strongly agreed that the podcasts with other health professionals enhanced their learning, and 98% agreed or strongly agreed that the live session with these health professionals further enhanced their learning. These reactions (New World Kirkpatrick level 1[22]) suggest successful achievement of Educational Objective 2 (educational value).

The submitted questions for the live portion of the workshop and the open-ended evaluation questions on the posttest captured additional New World Kirkpatrick level 2 (learning) evidence of the extent to which Educational Objectives were achieved.[22] Questions submitted by the students for the interprofessional interview session demonstrated both the desire to use respectful language in communicating with interprofessional colleagues (Educational Objective 3) and the value students placed on understanding the roles of other health professionals and how

Copyright © 2021 Miller et al. This is an open-access publication distributed under the terms of the Creative Commons Attribution license.

Table 1. Baseline Results of IPEC Competency Self-Assessment Tool Prior to Listening to Podcasts

Domain and Items	Agreement[a]	
	Percent	Number[b]
Interprofessional interaction domain		
I am able to choose communication tools and techniques that facilitate effective team interactions.	74	57
I am able to engage other health professionals in shared problem-solving appropriate to the specific care situation.	61	47
I am able to inform care decisions by integrating the knowledge and experience of other professions appropriate to the clinical situation.	61	47
I am able to apply leadership practices that support effective collaborative practice.	62	48
I am able to engage other health professionals to constructively manage disagreements about patient care.	52	40
I am able to use strategies that improve the effectiveness of interprofessional teamwork and team-based care.	58	45
I am able to use available evidence to inform effective teamwork and team-based practices.	73	56
I am able to understand the responsibilities and expertise of other health professions.	66	51
Overall	63	
Interprofessional values domain		
I am able to place the interests of patients at the center of interprofessional health care delivery.	94	72
I am able to respect the privacy of patients while maintaining confidentiality in the delivery of team-based care.	78	60
I am able to embrace the diversity that characterizes the health care team.	92	71
I am able to respect the cultures and values of other health professions.	94	72
I am able to develop a trusting relationship with other team members.	83	64
I am able to demonstrate high standards of ethical conduct in my contributions to team-based care.	88	68
I am able to act with honesty and integrity in relationships with other team members.	96	74
I am able to maintain competence in my own profession appropriate to my level of training.	87	67
Overall	89	

Abbreviation: IPEC, Interprofessional Education Collaborative.
[a]Students who selected *Agree* or *Strongly Agree*.
[b]Out of 77 total.

to leverage these various roles for team-based patient care (Educational Objective 1). Representative questions submitted by students are included in Table 4.

Prior to the workshop, we aimed for students' key learning takeaway to be strategies for engaging in productive, educational conversations with interprofessional colleagues (Educational Objective 4). Such strategies were explicitly mentioned in 22 out of 39 responses (56%). Other desired takeaways touched on learning more about the roles and responsibilities of specific professions (Educational Objective 1), valuing the role of other health professionals in medical education (Educational Objective 2), and the importance of respectful language in collaboration with interprofessional colleagues (Educational Objective 3); all three were evident in the responses of students. Illustrative quotations reflecting each Educational Objective are included in Table 4.

Table 2. Podcast Selection and Viewing ($N = 80$)

Interviewee Profession	Unique Views	Unique Students	Average Minutes Delivered	Length (Minutes)
Nurse practitioner	64	49	13.6	23.7
Occupational therapist	68	47	11.4	25.7
Social worker	113	64	14.6	38.1
Speech-language pathologist	48	35	13.0	27.9

Discussion

Our results demonstrate the value of podcasts as a medium for supporting medical students in engaging in dialogue with and learning from interprofessional colleagues. We successfully integrated podcasting to facilitate IPE and promote three of the four IPEC competencies. This workshop can be adapted for a variety of interprofessional learner populations and enacted with any health professionals as the featured podcast interviewees and panelists.

Medical student participants agreed or strongly agreed that the interprofessional representation in the podcasts and in the virtual follow-up enhanced their learning. This finding has been previously demonstrated using an interprofessional team-based approach,[13,23] but this curriculum is distinct in that it was conducted entirely virtually and incorporated other health professionals in the asynchronous and live portions of the experience. The virtual format helped to overcome some of the structural scheduling difficulties noted by other IPE curricula, including identifying and recruiting participants across health professions and the logistics involved in bringing panelists and students together.[9,13]

Podcasts provided additional advantages, including facilitating the availability of more professionals and allowing for more in-depth one-on-one conversations. Podcasts allow for flexibility

Copyright © 2021 Miller et al. This is an open-access publication distributed under the terms of the Creative Commons Attribution license.

Table 3. Changes in IPEC Competency Self-Assessment Domain Scores

	All Respondents[a]			Students With Paired Pretest and Posttest[b]		
**Domain[c]**	**Pretest *M* (*SD*)**	**Posttest *M* (*SD*)**	***p*[d]**	**Pretest *M* (*SD*)**	**Posttest *M* (*SD*)**	***p*[e]**
Interprofessional interaction	3.7 (0.6)	4.3 (0.5)	<.0001	3.8 (0.4)	4.3 (0.4)	<.0001
Interprofessional values	4.3 (0.5)	4.6 (0.4)	.0002	4.3 (0.4)	4.6 (0.4)	<.0001

Abbreviation: IPEC, Interprofessional Education Collaborative.
[a]Pretest $N = 77$, posttest $N = 39$.
[b]$N = 31$.
[c]Rated on a 5-point scale (1 = *Strongly Disagree*, 5 = *Strongly Agree*).
[d]Unpaired *t* test.
[e]Paired *t* test.

in recording and viewing, which enabled faculty to facilitate longer and broader extracurricular exposure to other health professionals. Other studies have documented the benefits of medical podcasts for other topics and their effectiveness for extracurricular knowledge acquisition.[24-27] Our data show that students accessed podcasts repeatedly and listened for brief periods. This suggests that students may have been sampling podcasts to determine which was most interesting or returning to a selected podcast to listen over multiple sessions. Furthermore, podcasts can be leveraged for students unable to participate in a live session or for a new cohort of students, either by allowing them to view a recording of the live session asynchronously or by scheduling a separate live session. Plans are already in place to use these podcasts for

Table 4. Student Questions and Responses to Educational Objectives

**Educational Objective**	**Posttest Responses: No. (%)[a]**	**Illustrative Quotations**
1. Roles and responsibilities	6 (15)	Questions submitted in live panel:<br>• "What are some specific privileges that advanced practice nurses have over other nurses? What are specific limitations they have?"<br>• "How much do [speech-language pathologists] overlap/collaborate specifically with [gastroenterology] services (e.g. swallowing disorders, nutrition)?"<br>• "For different specialties, when should we consult you about a patient?"<br>• "What can medical students do to advocate for expanded scope of practice for [nurse practitioners]?"<br>Responses submitted in posttest:<br>• "[I learned] just how complicated it can be when we don't understand the roles of other people in the care system."<br>• "I learned a lot about the perspectives/roles of different team members in the clinical space and the importance of communication and collaboration with various health professionals in providing patient care. Really fantastic session."
2. Educational value	4 (10)	Responses submitted in posttest:<br>• "Interprofessional members of the team have expertise in their field, I should listen to their advice rather than assume I know what the right thing to do."<br>• "You can always learn something new—never pass up an opportunity to learn from someone in another health profession, especially when they are working up a patient whose team you are also on."
3. Respectful language	7 (18)	Questions submitted in live panel:<br>• "What are the respectful ways of referring to other team members in various roles (e.g. for MDs Dr. Lastname, so for [doctors of nursing practice], Dr. Lastname? [registered nurses,] Nurse Lastname?)"<br>• "What do we do when the nurse knows more than we do, but we are expected to 'lead' the team (as a medical student, intern, or resident)?"<br>Responses submitted in posttest:<br>• "I liked the emphasis on respecting other professions when they're in the room with a patient. It is too easy to think you're the only busy one and that your work is most important."<br>• "One general theme I learned was to approach interactions with curiosity and respect, and that will get the best results."<br>• "The importance of not interrupting other providers as they are speaking with patients, as there should be no medical hierarchy."
4. Interprofessional communication strategies	22 (56)	Responses submitted in posttest:<br>• "I learned practical tips about asking interprofessional colleagues to help me learn more about high quality patient care."<br>• "Being humble and staying curious is probably the most important thing to do to help the collaboration."<br>• "Always introduce yourself."<br>• "Approaching others with curiosity and respect is the best way to establish a positive working relationship."

[a]Out of 39 total.

Copyright © 2021 Miller et al. This is an open-access publication distributed under the terms of the Creative Commons Attribution license.

a separate cohort of preclerkship students with a new live panel.

Several logistic considerations arose during the development, implementation, and evaluation. Facilitators had to identify interview questions of optimal relevance to the medical students. This required intentional inclusion of medical students in the interview process, as well as the creation of a mechanism for students to submit and curate follow-up questions so that medical students in the audience could engage in dialogue with the interviewees. Finally, students were given the opportunity to submit additional questions in real time as new points of interest arose during the live discussion.

An important consideration regarding the videoconferencing platform was ensuring a smooth session when podcast interviewees and facilitators were remote from one another. To ensure that the invited interprofessional colleagues focused on the intended learning outcomes, the detailed facilitator guide (Appendix F) included anticipated questions for podcast interviewees and was shared before the session. The facilitators were remote and had a separate method of communication via group text to ensure that they were coordinated while running the session, monitoring the Chat box, and maintaining the planned time line.

Limitations

While the Zoom platform allowed for the inclusion of interprofessional faculty, learners were limited to medical students, with no learners from other health professions. This was due to scheduling constraints of the medical school curriculum, and other interprofessional learners could be included in future iterations of the curriculum. In addition, not all students completed the pretest and posttest, introducing the potential for responder bias. Many students had left the Zoom conference prior to the posttest link appearing, which may explain the lower response rate for the posttest. As a result, matched self-assessment data were available for only 31 students despite a reminder email. However, pretest and posttest data were similar for this matched group and the overall cohort of respondents. Finally, a survey-based assessment limits the focus of the evaluation to New World Kirkpatrick levels 1 and 2.[20,21] In addition, survey-based data are subject to social desirability bias. To minimize this, all submitted questions and assessment responses were anonymous.

Future Directions

Future work will focus on recruiting interprofessional students to participate in asynchronous and synchronous learning sessions and on making the podcasts available beyond the scope of our course. To that end, we publish the podcasts here so that they can be used as stand-alone content or in conjunction with live conversations about themes related to IPE. Efforts are underway to have students document the conversations they engage in using the Student Framework card during their clinical rotations and to debrief these with faculty. It would also be helpful to understand how, when, and why and students listen to these podcasts. Understanding the reasons behind the observed use patterns could identify ways to increase engagement, such as introducing new professionals, modifying the interview format, adjusting length, or incorporating student voices into the podcasts. Importantly, this experience capitalized on the advantages of virtual platforms, including podcasts and videoconferencing, to facilitate interprofessional learning. These are important tools during the current pandemic and will remain valuable when future public health guidelines allow a return to in-person learning. The benefits of these platforms for IPE suggest that they should remain important tools regardless of the ability to engage in more traditional learning environments.

Appendices

A. Podcast Interview Guide.docx

B. Podcast - Nurse Practitioner.mp3

C. Podcast - Occupational Therapist.mp3

D. Podcast - Social Worker.mp3

E. Podcast - Speech-Language Pathologist.mp3

F. Facilitator Guide.docx

G. IPEC Competency Self-Assessment.docx

H. Interprofessional Clinical Conversations Framework.pptx

All appendices are peer reviewed as integral parts of the Original Publication.

Kelsey A. Miller, MD, EdM: Instructor, Pediatrics, Harvard Medical School; ORCID: https://orcid.org/0000-0002-0323-2048

Tamra Keeney, DPT, PhD: Instructor, Medicine, Harvard Medical School; ORCID: https://orcid.org/0000-0002-2603-8707

Allison Fialkowski, BSN: Third-Year Medical Student, Harvard Medical School; ORCID: https://orcid.org/0000-0002-1314-923X

Sanjana Srinivasan: First-Year Medical Student, Harvard Medical School

Tara A. Singh, MD: Instructor, Obstetrics, Gynecology and Reproductive Biology, Harvard Medical School; ORCID: https://orcid.org/0000-0003-3210-4800

Copyright © 2021 Miller et al. This is an open-access publication distributed under the terms of the Creative Commons Attribution license.

Jennifer Kesselheim, MD, MEd: Associate Professor, Pediatrics, Harvard Medical School; ORCID: https://orcid.org/0000-0002-5765-9813

Susan Farrell, MD, EdM: Associate Professor, Emergency Medicine, Harvard Medical School

Cynthia Cooper, MD: Assistant Professor, Medicine, Harvard Medical School; ORCID: https://orcid.org/0000-0002-6062-4339

Celeste S. Royce, MD: Assistant Professor, Obstetrics, Gynecology and Reproductive Biology, Harvard Medical School; ORCID: https://orcid.org/0000-0002-0739-0174

Acknowledgments
Kelsey A. Miller and Tamra Keeney contributed equally to this work and are co-primary authors.

Disclosures
None to report.

Funding/Support
None to report.

Ethical Approval
Reported as not applicable.

References

1. Anderson ES, Gray R, Price K. Patient safety and interprofessional education: a report of key issues from two interprofessional workshops. *J Interprof Care*. 2017;31(2):154-163. https://doi.org/10.1080/13561820.2016.1261816
2. Oxelmark L, Nordahl Amorøe T, Carlzon L, Rystedt H. Students' understanding of teamwork and professional roles after interprofessional simulation—a qualitative analysis. *Adv Simul (Lond)*. 2017;2:8. https://doi.org/10.1186/s41077-017-0041-6
3. Institute of Medicine. *To Err Is Human: Building a Safer Health System*. National Academies Press; 2000. https://doi.org/10.17226/9728
4. Institute of Medicine. *Crossing the Quality Chasm: A New Health System for the 21st Century*. National Academies Press; 2001. https://doi.org/10.17226/10027
5. National Academies of Sciences, Engineering, and Medicine. *Improving Diagnosis in Health Care*. National Academies Press; 2015. https://doi.org/10.17226/21794
6. Liaison Committee on Medical Education. *Functions and Structure of a Medical School: Standards for Accreditation of Medical Education Programs Leading to the MD Degree*. Liaison Committee on Medical Education; 2020.
7. Interprofessional Education Collaborative. *Core Competencies for Interprofessional Collaborative Practice: 2016 Update*. Interprofessional Education Collaborative; 2016. https://ipec.memberclicks.net/assets/2016-Update.pdf
8. *Core Entrustable Professional Activities for Entering Residency: Curriculum Developers' Guide*. Association of American Medical Colleges; 2014. https://store.aamc.org/downloadable/download/sample/sample_id/63/
9. Lawlis TR, Anson J, Greenfield D. Barriers and enablers that influence sustainable interprofessional education: a literature review. *J Interprof Care*. 2014;28(4):305-310. https://doi.org/10.3109/13561820.2014.895977
10. Sterling M, Leung P, Wright D, Bishop TF. The use of social media in graduate medical education: a systematic review. *Acad Med*. 2017;92(7):1043-1056. https://doi.org/10.1097/ACM.0000000000001617
11. Berk J, Trivedi SP, Watto M, Williams P, Centor R. Medical education podcasts: where we are and questions unanswered. *J Gen Intern Med*. 2020;35(7):2176-2178. https://doi.org/10.1007/s11606-019-05606-2
12. Greenfield S. Podcasting: a new tool for student retention? *J Nurs Educ*. 2011;50(2):112-114. https://doi.org/10.3928/01484834-20101230-06
13. Kesselheim JC, Stockman LS, Growdon AS, Murray AM, Shagrin BS, Hundert EM. Discharge Day: a case-based interprofessional exercise about team collaboration in pediatrics. *MedEdPORTAL*. 2019;15:10830. https://doi.org/10.15766/mep_2374-8265.10830
14. Sanders M, Richards D, Panahi L. Interprofessional education (IPE) in a pediatric simulation: case of an infant with fever. *MedEdPORTAL*. 2013;9:9515. https://doi.org/10.15766/mep_2374-8265.9515
15. Tegzes J, Mackintosh S, Meyer T, Redman-Bentley D, Aston S. To be or not to be: an interprofessional problem-based learning case introducing the One Health Initiative. *MedEdPORTAL*. 2013;9:9623. https://doi.org/10.15766/mep_2374-8265.9623
16. Lairamore C, George-Paschal L, McCullough K, Grantham M, Head D. A case-based interprofessional education forum improves students' perspectives on the need for collaboration, teamwork, and communication. *MedEdPORTAL*. 2013;9:9484. https://doi.org/10.15766/mep_2374-8265.9484
17. Ro K, Villarreal J. Interprofessional pharmacology podcasting in advanced practice nursing seminar. *Nurse Educ*. 2019;44(5):237-238. https://doi.org/10.1097/NNE.0000000000000636
18. Poirier TI, Stamper-Carr C, Newman K. A course for developing interprofessional skills in pre-professional honor students using humanities and media. *Curr Pharm Teach Learn*. 2017;9(5):874-880. https://doi.org/10.1016/j.cptl.2017.05.004
19. Milles LS, Hitzblech T, Drees S, Wurl W, Arends P, Peters H. Student engagement in medical education: a mixed-method study on medical students as module co-directors in curriculum development. *Med Teach*. 2019;41(10):1143-1150. https://doi.org/10.1080/0142159X.2019.1623385
20. Dow AW, DiazGranados D, Mazmanian PE, Retchin SM. An exploratory study of an assessment tool derived from the

Copyright © 2021 Miller et al. This is an open-access publication distributed under the terms of the Creative Commons Attribution license.

competencies of the Interprofessional Education Collaborative. *J Interprof Care*. 2014;28(4):299-304. https://doi.org/10.3109/13561820.2014.891573

21. Lockeman KS, Dow AW, Randell AL. Validity evidence and use of the IPEC Competency Self-Assessment, Version 3. *J Interprof Care*. 2021;35(1):107-113. https://doi.org/10.1080/13561820.2019.1699037
22. Kirkpatrick JD, Kirkpatrick WK. *Kirkpatrick's Four Levels of Training Evaluation*. ATD Press; 2016.
23. Marks A, Hollenkamp E, Bradman S, et al. A child's last hours—multidisciplinary training in end-of-life care in children's hospitals: school-aged child with terminal cancer. *MedEdPORTAL*. 2016;12:10371. https://doi.org/10.15766/mep_2374-8265.10371
24. Lien K, Chin A, Helman A, Chan TM. A randomized comparative trial of the knowledge retention and usage conditions in undergraduate medical students using podcasts and blog posts. *Cureus*. 2018;10(1):e2065. https://doi.org/10.7759/cureus.2065
25. Chin A, Helman A, Chan T. Podcast use in undergraduate medical education. *Cureus*. 2017;9(12):e1930. https://doi.org/10.7759/cureus.1930
26. Malecki SL, Quinn KL, Zilbert N, et al. Understanding the use and perceived impact of a medical podcast: qualitative study. *JMIR Med Educ*. 2019;5(2):e12901. https://doi.org/10.2196/12901
27. Chan TM, Dzara K, Dimeo SP, Bhalerao A, Maggio LA. Social media in knowledge translation and education for physicians and trainees: a scoping review. *Perspect Med Educ*. 2020;9(1):20-30. https://doi.org/10.1007/s40037-019-00542-7

Received: February 26, 2021
Accepted: August 2, 2021
Published: October 25, 2021

Copyright © 2021 Miller et al. This is an open-access publication distributed under the terms of the Creative Commons Attribution license.
